# Combined non-surgical treatment for Paget–Schröetter syndrome: a case report

**DOI:** 10.1186/s13256-016-0940-5

**Published:** 2016-06-10

**Authors:** Gemma Edo Fleta, Álvaro Torres Blanco, Francisco Gómez Palonés, Eduardo Ortiz Monzón

**Affiliations:** Department of Angiology, Vascular and Endovascular Surgery, Hospital Universitario Doctor Peset, Avenida de Gaspar Aguilar, 90, 46017 Valencia, Spain

**Keywords:** Paget–Schröetter syndrome, Thrombosis, Thrombolysis, Anticoagulation, Thoracic outlet

## Abstract

**Background:**

Paget–Schröetter syndrome is an uncommon form of venous thrombosis, which is related to thoracic outlet syndrome. Axillary-subclavian vein thrombosis typically presents in healthy young adults.

We present this case of particular interest because it indicates that a combined treatment involving thrombolysis, anticoagulation therapy, rehabilitation, and elastic compression sleeves can be a valid non-surgical alternative for some patients with Paget–Schröetter syndrome.

**Case presentation:**

This report describes a case of a 38-year-old white woman, a swimmer, who presented with a sudden episode of swelling and pain in her right upper extremity. After duplex ultrasound diagnosis of venous thrombosis, computed tomography (CT) showed extrinsic compression of the vessel. Catheter-directed thrombolysis was performed in the first 24 hours, followed by anticoagulant therapy with bemiparin at a dose of 7500 IU/24 hours for the first week, and then reduced to 3500 IU/24 hours for the next 3 months. After treatment there was restoration of her venous flow and she returned to work 2 weeks later. Anticoagulant treatment was continued for 3 months; decompression surgery was not performed. At 6 months she was asymptomatic.

**Conclusion:**

Combined treatment involving thrombolysis, anticoagulant therapy, rehabilitation, and elastic compression sleeves may be a valid non-surgical alternative for a selected subset of patients with Paget–Schröetter syndrome.

## Background

Paget–Schröetter syndrome (PSS) or “effort” axillary-subclavian vein thrombosis is a relatively uncommon condition that affects young, active, healthy individuals [1]. This clinical entity has been related to bony, ligamentous, and muscular abnormalities that can cause compression of the subclavian vein where it passes between the clavicle and the first rib [2, 3]. Some individuals may present a congenital narrowing of the thoracic outlet, while others can develop this narrowing as a result of vigorous activity or exercise, resulting in muscle hypertrophy. Clinical features are variable, from asymptomatic cases to the possibility of presenting pain, swelling, and profuse collateral circulation.

At present there is no consensus on the best management for PSS. Early treatment is recommended [4, 5] and a multifaceted approach appears to be the best option: the most widely regarded management strategies involve a combination of catheter-directed thrombolysis and surgical decompression, although a minority prefers a non-surgical approach. In fact, thoracic outlet decompression usually is achieved by removal of the first rib, but this surgical procedure can be associated with complications. In contrast, anticoagulation therapy is a non-surgical option, with a lower risk and can give good results in selected patients. Here we describe a case of a woman who was a keen *amateur* swimmer who presented with PSS that was successfully managed with combined non-surgical treatment.

## Case presentation

### Personal and family history of the patient

A 38-year-old white woman presented with swelling and pain in her right upper limb that had begun 48 hours previously and that was unrelated to trauma. She was a keen *amateur* swimmer and had increased training in the days before the onset of the swelling. She had a history of bilateral shoulder recurrent luxation during childhood, which led to an operation on her left shoulder when she was 24-years old. Her history did not reveal any risk factors for venous thromboembolic disease (VTE): she had not recently travelled or had prolonged immobilization, nor had there been any recent surgery (the shoulder surgery occurred 14 years prior and was on her left shoulder, which was not the site of the swelling and pain that precipitated this case). Furthermore, she had no family history of thromboembolic disease or thrombophilia and was not taking oral contraceptive pills. The only historical detail of interest was bilateral recurrent shoulder dislocation in childhood, although there was no episode prior to the presentation of this PSS.

### Physical examination and other tests

**Fig. 1:**
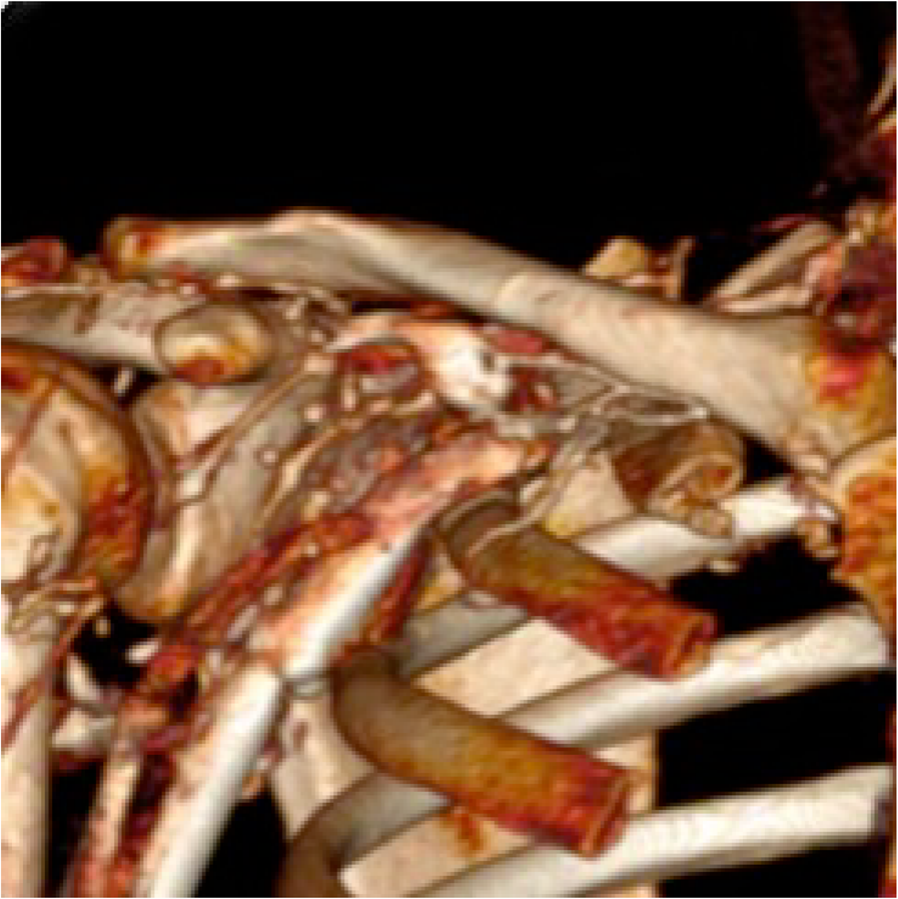
Computed tomography angiography scan of right shoulder

There were no signs of arterial disease; humeral, radial, and ulnar pulses on her upper limbs were present and good capillary filling was observed without skin changes. An examination revealed an increased diameter of her right upper limb, as well as an edema in all her right upper limb and deltopectoral collateral circulation. Upper limb mobility and sensitivity were normal and preserved, without paresthesia or dysesthesia that could be suspicious for nerve or arterial compression. Following the protocol of our center, ultrasound tests were performed to detect deep venous thrombosis (DVT), to evaluate compression, occupation of light, and color flow, and Doppler ultrasound was used to assess the phasic flow. The duplex ultrasound revealed a lack of compressibility, permeability, and phasic flow in the middle third and proximal region of her subclavian vein; right subclavian vein thrombosis was diagnosed. Computed tomography angiography (CTA) of her supra-aortic trunks, thorax, and upper extremities confirmed thrombosis in her right subclavian vein, just below her collarbone and her first rib (Fig. 1). Multiple collateral veins in her right upper limb were observed, which enlarged the limb compared with her contralateral limb. No other findings of interest were noted.

### Treatment and follow-up

**Fig. 2:**
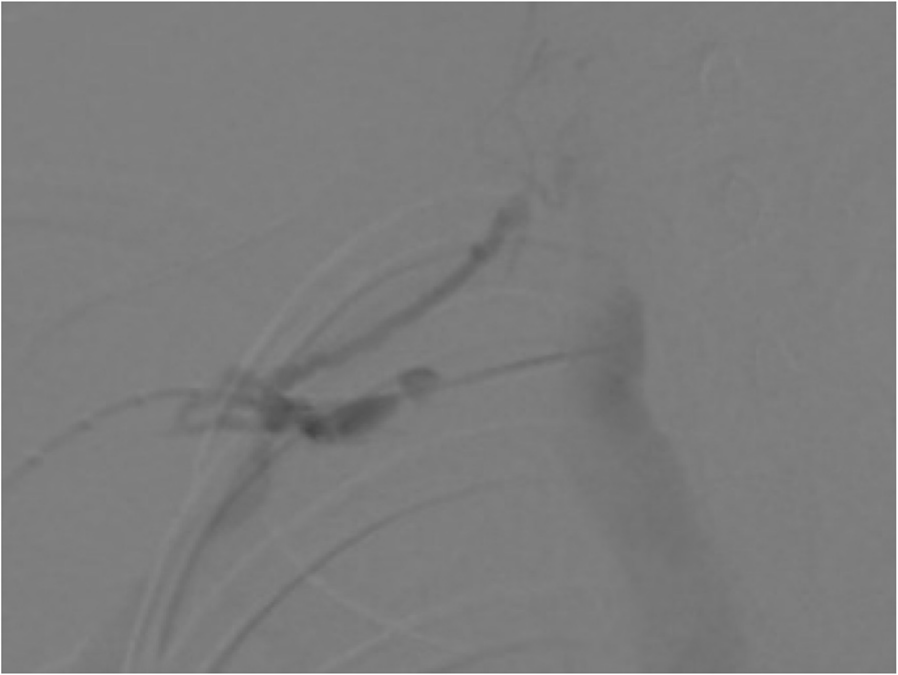
Venography confirmed thrombosis, a profuse collateral network, and proximal subclavian vein patency

Venography was performed via her right cephalic vein, confirming thrombosis, a profuse collateral network, and proximal subclavian vein patency (Fig. 2). With a multi-side-hole catheter placed in the thrombus, a 250,000 IU urokinase bolus was administered for local fibrinolysis, followed by a continuous perfusion of 100,000 IU/hour for 24 hours.

**Fig. 3:**
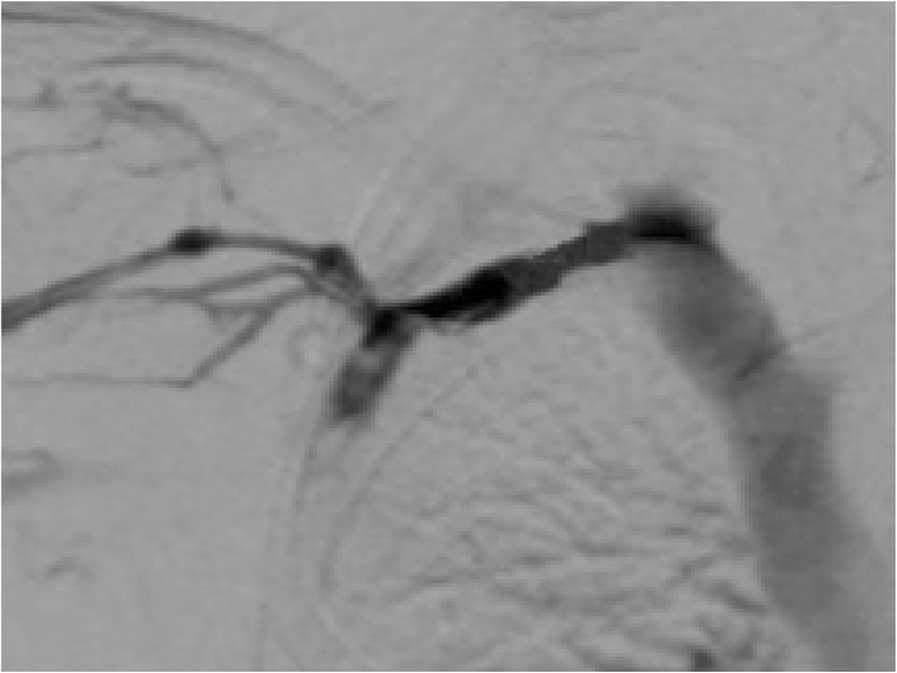
Venography 24 hours later showing partial recanalization of the thrombus and a persisting moderate residual stenosis

After 24 hours, venography showed partial recanalization of the thrombus and a persisting moderate residual stenosis (Fig. 3). Percutaneous transluminal angioplasty of the stenosis was performed with a 6×40 mm balloon.

After a further 24 hours she was showing clinical improvement and was discharged. During the first week ambulatory treatment with low molecular weight heparin (LMWH) was provided at a therapeutic dose; for the subsequent 3 months ambulatory treatment comprised prophylactic doses of LMWH together with an elastic compression sleeve and physiotherapy. She was advised on preventive measures; she was recommended to abstain from exercises involving the upper extremity and swimming (identified as the precipitant element). This was combined with in-hospital and at-home physiotherapy, which provided advice and education on exercise and lifestyle modifications.

She was followed-up via ambulatory consultation (after 15 days, 1 month, 3, 6 and 12 months, and then yearly) to monitor her return to normal/working life and physical condition (including eco-Doppler). At the 6-month follow-up, the permeability of her subclavian vein was examined by ultrasound: clinical improvement had been maintained with no swelling and no functional impact; duplex ultrasound demonstrated subclavian vein patency.

## Discussion

In 1875 Paget described subclavian vein thrombosis and in 1884 Schröetter proposed that subclavian vein thrombosis was the result of excessive upper extremity activity. In 1949, the surgeon Hughes coined the term “Paget–Schröetter syndrome” for patients with occlusion of the subclavian vein following overuse of the upper extremity [1, 3, 6].

DVT occurs more commonly in the veins of the pelvis or the lower extremities. In two previous reports, the incidence of upper extremity DVT has been cited as occurring in approximately 4 to 11 % of all cases of thromboembolism. DVT is classified as either primary or secondary, depending of the cause of the thrombus. Primary or idiopathic upper extremity DVT is less common than secondary, only accounting for 2 in 100,000 cases per year [7, 8]. Secondary DVT is more common and can be caused by central venous catheters, cardiac devices, neoplasias, or collagen diseases. Thrombosis of the upper extremity veins is usually related to effort in conjunction with anatomical abnormalities of the thoracic outlet. Some individuals present congenital narrowing of the thoracic outlet, which could lead to an increased risk of developing thrombosis. Another hypothesis is that vigorous activity or exercise could result in an anterior scalene or subclavius muscle hypertrophy that could lead to compression of the vessel over the first rib [7]. It has been also reported in some cases that the first rib exhibits an abnormal bony tubercle just lateral to the costosternal joint [3]. In all these conditions, the theory for thrombus formation is the repetitive compression of the vessel, which may lead to a microvascular trauma and sometimes to venous fibrosis and activation of the coagulation cascade [7]. Most patients refer a precipitant event, usually sport or exercise, or as a result of occupational exertion [1, 8].

The gold standard for diagnosis of PSS is catheter-directed contrast venography, although the first-line imaging method is usually duplex ultrasound. The sensitivity and specificity of ultrasound with color Doppler to diagnose upper extremity DVT have been reported as 70 to 100 % and 93 %, respectively. Magnetic resonance and CT scanning can be used for the assessment of the thoracic outlet.

There is no consensus on the best treatment for PSS. Thrombolytic therapy in the acute phase is very effective and it is the treatment of choice. After catheter-directed thrombolysis, some authors suggested that the surgical decompression of the thoracic outlet is a necessary part of treatment of most patients. The best time to perform it is unclear; while some recommended acting immediately in order to avoid the risk of recurrence, others preferred to perform surgical decompression 3 months later, as this can reduce the rate of complications [5, 9]. The most commonly used technique is the removal of the first rib through a transaxillary approach. The main drawback of surgery is the risk of complications such as pneumothorax, pleural effusion, thoracic duct lymph leak, brachial plexus, and phrenic nerve or long thoracic nerve injury/dysfunction. In general, angioplasty or stenting are not recommended in the case of extrinsic compression, as these techniques do not resolve the extrinsic compression and may generate endothelial injury [10]. Given the moderate grade of residual stenosis and the agreement of the patient to perform rehabilitation exercises, we decided not to perform surgical intervention for decompression, maintaining only the anticoagulant therapy, which is recommended for 3 to 6 months. Maintenance treatment with a prophylactic dose of LMWH can provide a clinical benefit with a low hemorrhagic risk.

## Conclusions

Although PSS management with fibrinolysis and decompression surgery is an option with good results, fibrinolysis and anticoagulant therapy could be a less invasive alternative in selected cases. Based on our experience, we propose that the less invasive treatment described here is suitable for patients with only venous involvement; however, for patients with compromised nervous or hematological aspects, we would consider more aggressive treatment with decompression surgery.

Studies with more patients and long-term follow-up are necessary to assess the possibility of recurrence.

## Abbreviations

CT, computed tomography; CTA, computed tomography angiography; DVT, deep venous thrombosis; LMWH, low molecular weight heparin; PSS, Paget–Schröetter syndrome; VTE, venous thromboembolic disease
